# VarCards2: an integrated genetic and clinical database for ACMG-AMP variant-interpretation guidelines in the human whole genome

**DOI:** 10.1093/nar/gkad1061

**Published:** 2023-11-13

**Authors:** Zheng Wang, Guihu Zhao, Zhaopo Zhu, Yijing Wang, Xudong Xiang, Shiyu Zhang, Tengfei Luo, Qiao Zhou, Jian Qiu, Beisha Tang, Kun Xia, Bin Li, Jinchen Li

**Affiliations:** National Clinical Research Center for Geriatric Disorders, Department of Geriatrics, Xiangya Hospital, Central South University, Changsha, Hunan 410008, China; Department of Neurology, Xiangya Hospital, Central South University, Changsha, Hunan 410008, China; Hunan Key Laboratory of Molecular Precision Medicine, Xiangya Hospital, Central South University, Changsha, Hunan 410008, China; National Clinical Research Center for Geriatric Disorders, Department of Geriatrics, Xiangya Hospital, Central South University, Changsha, Hunan 410008, China; Department of Neurology, Xiangya Hospital, Central South University, Changsha, Hunan 410008, China; Bioinformatics Center, Furong Laboratory & Xiangya Hospital, Central South University, Changsha, Hunan 410008, China; Center for Medical Genetics & Hunan Key Laboratory, School of Life Sciences, Central South University, Changsha, Hunan 410008, China; National Clinical Research Center for Geriatric Disorders, Department of Geriatrics, Xiangya Hospital, Central South University, Changsha, Hunan 410008, China; Bioinformatics Center, Furong Laboratory & Xiangya Hospital, Central South University, Changsha, Hunan 410008, China; National Clinical Research Center for Geriatric Disorders, Department of Geriatrics, Xiangya Hospital, Central South University, Changsha, Hunan 410008, China; Xiangya School of Medicine, Central South University, Changsha, Hunan 410013, China; Center for Medical Genetics & Hunan Key Laboratory, School of Life Sciences, Central South University, Changsha, Hunan 410008, China; National Clinical Research Center for Geriatric Disorders, Department of Geriatrics, Xiangya Hospital, Central South University, Changsha, Hunan 410008, China; Bioinformatics Center, Furong Laboratory & Xiangya Hospital, Central South University, Changsha, Hunan 410008, China; National Clinical Research Center for Geriatric Disorders, Department of Geriatrics, Xiangya Hospital, Central South University, Changsha, Hunan 410008, China; Department of Neurology, Xiangya Hospital, Central South University, Changsha, Hunan 410008, China; Hunan Key Laboratory of Molecular Precision Medicine, Xiangya Hospital, Central South University, Changsha, Hunan 410008, China; National Clinical Research Center for Geriatric Disorders, Department of Geriatrics, Xiangya Hospital, Central South University, Changsha, Hunan 410008, China; Department of Neurology, Xiangya Hospital, Central South University, Changsha, Hunan 410008, China; Department of Neurology, & Multi-Omics Research Center for Brain Disorders, The First Affiliated Hospital, University of South China, Hengyang, Hunan, China; Center for Medical Genetics & Hunan Key Laboratory, School of Life Sciences, Central South University, Changsha, Hunan 410008, China; National Clinical Research Center for Geriatric Disorders, Department of Geriatrics, Xiangya Hospital, Central South University, Changsha, Hunan 410008, China; Department of Neurology, Xiangya Hospital, Central South University, Changsha, Hunan 410008, China; Bioinformatics Center, Furong Laboratory & Xiangya Hospital, Central South University, Changsha, Hunan 410008, China; National Clinical Research Center for Geriatric Disorders, Department of Geriatrics, Xiangya Hospital, Central South University, Changsha, Hunan 410008, China; Center for Medical Genetics & Hunan Key Laboratory, School of Life Sciences, Central South University, Changsha, Hunan 410008, China; Department of Neurology, Xiangya Hospital, Central South University, Changsha, Hunan 410008, China; Bioinformatics Center, Furong Laboratory & Xiangya Hospital, Central South University, Changsha, Hunan 410008, China

## Abstract

VarCards, an online database, combines comprehensive variant- and gene-level annotation data to streamline genetic counselling for coding variants. Recognising the increasing clinical relevance of non-coding variations, there has been an accelerated development of bioinformatics tools dedicated to interpreting non-coding variations, including single-nucleotide variants and copy number variations. Regrettably, most tools remain as either locally installed databases or command-line tools dispersed across diverse online platforms. Such a landscape poses inconveniences and challenges for genetic counsellors seeking to utilise these resources without advanced bioinformatics expertise. Consequently, we developed VarCards2, which incorporates nearly nine billion artificially generated single-nucleotide variants (including those from mitochondrial DNA) and compiles vital annotation information for genetic counselling based on ACMG-AMP variant-interpretation guidelines. These annotations include (I) functional effects; (II) minor allele frequencies; (III) comprehensive function and pathogenicity predictions covering all potential variants, such as non-synonymous substitutions, non-canonical splicing variants, and non-coding variations and (IV) gene-level information. Furthermore, VarCards2 incorporates 368 820 266 documented short insertions and deletions and 2 773 555 documented copy number variations, complemented by their corresponding annotation and prediction tools. In conclusion, VarCards2, by integrating over 150 variant- and gene-level annotation sources, significantly enhances the efficiency of genetic counselling and can be freely accessed at http://www.genemed.tech/varcards2/.

## Graphical Abstract

**Graphical Abstract:**
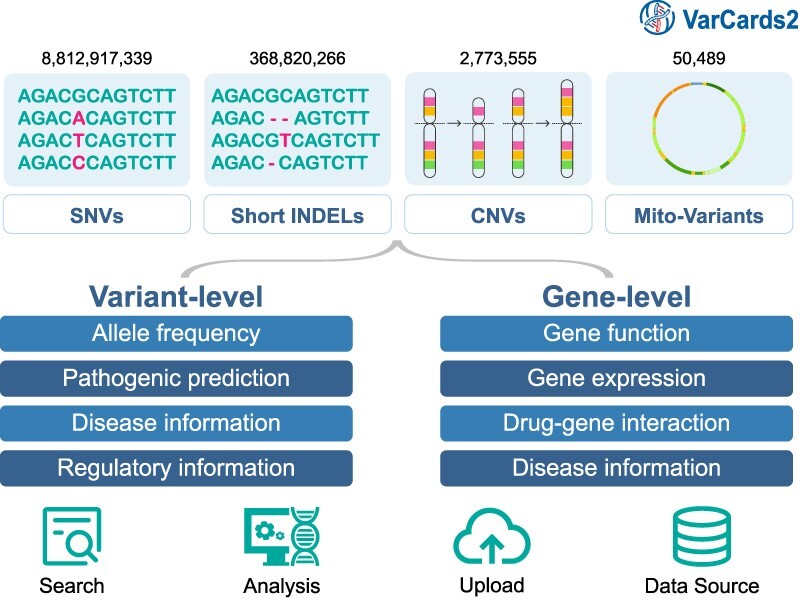

## Introduction

Rapid advances in sequencing technology over the last few years have provided unprecedented opportunities and challenges for genetic counselling (1). To help clinicians and clinical laboratory geneticists address new challenges in sequence interpretation, standards and guidelines for interpreting sequence variants were developed by the American College of Medical Genetics and Genomics (ACMG) (2). It is well established that these standards and guidelines from ACMG are the best practices for genetic counselling. However, most datasets and *in silico* algorithms recommended by the ACMG for sequence variant interpretation are dispersed across various online platforms and databases. In response, we introduced VarCards (http://www.genemed.tech/varcards/), a comprehensive online database, to equip users with essential genetic and clinical knowledge for genetic counselling on specific coding variants (3). Because VarCards streamlines genetic counselling by offering gene- and variant-level annotation information recommended by the ACMG, VarCards has accessed more than 372 000 visits since its launch.

With the clinical significance of non-coding single-nucleotide variants (SNVs) and copy number variants (CNVs) of the human genome in genetic counselling raised more emphasis (4–8), a growing number of genomic tools or databases were developed to facilitate the interpretation of these non-coding variations (9–32). Still, they were either locally installed databases (such as GREEN-DB (9) and regBase (28)) or command-line tools (such as ClassifyCNV (33) and DIVAN (14)). In addition, many important annotation sources, such as allele frequencies, expression quantitative trait loci (eQTL), and regulatory information, have been dispersed across various online platforms. This widespread dispersion complicates the process for general clinicians, genetic counsellors and clinical laboratory geneticists trying to quickly access up-to-date data to interpret the function and pathogenicity of variations in the whole human genome in line with the standards and guidelines of the ACMG.

Although comprehensive human variation annotation databases, such as VARAdb (34) and VannoPortal (35) exist, their primary emphasis is on providing detailed data on regulatory profiles and evolutionary signatures. This is convenient for biologists to explore the underlying molecular mechanisms but does not specifically address the need for clinical genetic counselling. In addition, some of these databases, such as VARAdb (34), compiled a total of 577 283 813 variations, of which the majority were single-nucleotide polymorphisms (SNPs) with a low likelihood of pathogenicity; however, there should theoretically be nearly nine billion SNVs in the human genome. Moreover, these databases did not include CNVs or detailed gene-level annotations.

To support clinicians, genetic counsellors, and clinical laboratory geneticists in providing effective genetic counselling, we developed VarCards2, an intuitive online database. It houses nearly nine billion SNVs, over 360 million documented short insertions and deletions (INDELs), and more than two million CNVs. VarCards2 provides in-depth annotations at both the variant and gene levels, including *in silico* predictions of function and pathogenicity, minor allele frequencies (MAFs) across diverse populations, splicing predictions for both canonical and non-canonical splicing regions, and gene functionality, all in alignment with standards and guidelines from ACMG.

## Materials and methods

### Variant-level data source

To optimise the support for genetic counselling, VarCards2 encompassed nearly nine billion SNVs, representing any base in the human reference genome GRCh38 (including mitochondrial DNA) that had mutated into one of the three possible bases. Additionally, VarCards2 houses all reported short INDELs (length ≤ 50 bp) and CNVs (length > 50 bp) extracted from the subsequent seven databases: (I) the Genome Aggregation Database (gnomAD) (36); (II) the International Cancer Genome Consortium (ICGC) (37); (III) the clinical variations database (ClinVar) (38); (IV) the Catalogue of Somatic Mutations In Cancer (COSMIC) (39); (V) *de novo* mutations database called Gene4Denovo (40); (VI) the NCBI database of genetic variation named dbSNP (41) and (VII) the NCBI database of human genomic structural variation named dbVar (42).

We sourced allele frequency (AF) data for various ethnic backgrounds from several publicly accessible population databases, such as (I) gnomAD v2.1.1 (125748 exomes and 15 708 genomes) (43), (II) gnomAD v3.1.2 (76 156 genomes) (36), (III) the Exome Aggregation Consortium (ExAC) (60 706 exomes) (44), (IV) 1000 Genomes Project (2504 individuals genomic data) (45), (V) Exome Sequencing Project (ESP) (6503 exomes) (46), (VI) Kaviar genomic variant database (13200 genomes and 64600 exomes) (47), (VII) the Haplotype Reference Consortium (HRC) (64976 haplotypes) (48) and (VIII) a database for the human mitochondrial genome named MITOMAP (51836 full-length mitochondrial sequences) (49). Additionally, we retrieved information on variants and their associated diseases or phenotypes from ClinVar (38), ICGC (37), COSMIC (39), InterVar (50) and the NHGRI-EBI catalogue of human genome-wide association studies (GWAS) (51). Moreover, we extracted functional and pathogenicity prediction scores from more than 100 *in silico* algorithms and tools. These tools encompass 50 coding region SNVs, 24 non-coding region SNVs, 19 splice variants, 4 INDELs (52–55), 4 CNVs (33,56–58) and 25 mitochondrial DNA variants. In particular, the prediction scores of non-synonymous variants for coding regions were sourced from the dbNSFP v4.4 database (59,60), in addition to two recently introduced prediction tools: CAPICE and AlphaMissense (53,61); prediction scores for non-coding regions and splice variants were sourced from our previous studies (62,63); prediction score for mitochondrial DNA variants was sourced from a collection of genomic, clinical, and functional annotations for human mitochondrial DNA variants named MitImpact (64,65). Furthermore, some variant information, such as reported *de novo* mutations (40) and splice variants (63), and some regulatory information, including expression quantitative trait loci (eQTL) (66), splicing quantitative trait loci (sQTL) (66), summary data from VARAdb (34), GREEN-DB(9), and EPimap EPigenomics (67), were also catalogued (Table 1). Additionally, to offer a more intuitive and straightforward interface, we have visualized EpiMap Epigenomics data using heatmaps and furnished an additional panel named ‘Variant Summary’ employing the following measures: (I) The corresponding rsID for the variant and the link redirecting to the dbSNP database. (II) The corresponding positions in GRCh37 and GRCh38 for the variant, along with the link redirecting to the UCSC Genome Browser. (III) The corresponding amino acid change associated with the variant. (IV) A variant is designated as a 'rare variant' if its AF is below 0.1% in the gnomAD database, version 3.12. (V) Summarised information from ClinVar, including 'Clinical Significance’, ‘Review Status’ and 'Condition’, is displayed. (VI) If a variant is predicted to be deleterious by more than 60% of the prediction tools, it is considered putatively harmful.

**Table 1.** Summary of integrated data sources in VarCards2

Category	Data source
**Part one: variation-level implication**	
Allele frequency	gnomAD, ExAC, 1000 Genomes, ESP, Kaviar, HRC, Mitomap
In silico function and pathogenicity prediction	ReVe, CADD, DANN, Eigen, Fathmm-MKL, FATHMM, FitCons, GenoCanyon, REVEL, SIFT, PolyPhen2-HDIV, PolyPhen2-HVAR, LRT, MutationTaster, MutationAssessor, PROVEAN, VEST4, MetaSVM, MetaLR, M-CAP, GERP++, phyloP100way-vertebrate, phastCons100way-vertebrate, SiPhy, Eigen-PC, Fathmm-XF, SIFT4G, LINSIGHT, MutPred2, MVP, MPC, PrimateAI, DEOGEN2, BayesDel-addAF, BayesDel-noAF, ClinPred, LIST-S2, ALoFT, bStatistic, phyloP470way-mammal, phyloP17way-primate, phastCons470way-mammal, phastCons17way-primate, gMVP, VARITY-R, VARITY-ER, VARITY-R-LOO, VARITY-ER-LOO, AlphaMissense, FitCons2, Funseq2, ReMM, CScape, Orion, FIRE, PAFA, CDTS, DVAR, ncER, regBase-REG, regBase-CAN, regBase-PAT, Divan-TSS, Divan-Region, CADD-splice, SCAP, spliceAI, dpsi-max-tissue, dpsi-zscore, dbscSNV-ADA-SCORE, dbscSNV-RF-SCORE, MaxEntScan, GeneSplicer, ESRseq, Spliceogen, Squirl, RegSNPs-intron, MMSplice, KipoiSplice, Synvep, SPiCE-MES, SPiCE-SSF, SPiCE, CADD-SV, AnnotSV, ClassifyCNV, StrVCTVRE, FatHmmW, EFIN-SP, EFIN-HD, PANTHER, PhD-SNP, SNAP, Mitoclass1, SNPDryad, Meta-SNP, CAROL, Condel, COVEC-WMV, MtoolBox, APOGEE, MitoTIP, PON-Classification, CAPICE, FATHMM-indel, PROVEAN-indel
Disease-related	ClinVar, InterVar, ICGC, COSMIC, GWAS Catalog
Variant information	Gene4Denovo, SPCards
Regulatory information	GTEx, VARAdb, GREEN-DB, EPimap EPigenomics
**Part two: gene-level implication**	
Basic information	NCBI Gene, Entrez, OMIM, HGNC, Ensembl, GeneCards, UniProtKB
Genic intolerance	RVIS, LoFtool, GDI, Episcore, heptanucleotide context intolerance score, pLI score
Gene function	Gene Ontology, UniProtKB, InterPro, NCBI BioSystems, InBio Map™
Disease-related	OMIM, ClinVar, GeneReviews, ClinGen, Human Phenotype Ontology, GenCC, DECIPHER, Orpha data, DisGeNET, GTR, Noncode, MGI, Gene4Denovo
Gene expression	BrainSpan, GTEx, Allen Brain Atlases, The Human Protein Atlas
Target drug	DGIdb, PharmGKB, CTD, Drug Central, Drug Target Commons

*Note:* gnomAD, Genome Aggregation Database; ExAC, Exome Aggregation Consortium; 1000 Genomes, The 1000 Genomes Project; ESP, Exome Sequencing Project; Kaviar, Known VARiants; HRC, Haplotype Reference Consortium; Mitomap, A Human Mitochondrial Genome Database; CADD, Combined Annotation Dependent Depletion; DANN, Deep Neural Network-based Annotation; Fathmm-MKL, Functional Analysis Through Hidden Markov Models-Multitask Learning; FATHMM, Functional Analysis Through Hidden Markov Models; FitCons, Fitness Consequences; REVEL, Rare Exome Variant Ensemble Learner; SIFT, Sorting Intolerant From Tolerant; PolyPhen2-HDIV, Polymorphism Phenotyping v2 - HumDiv model; PolyPhen2-HVAR, Polymorphism Phenotyping v2 - HumVar model; LRT, Likelihood Ratio Test; PROVEAN, Protein Variation Effect Analyzer; ClinVar, Clinical Variation Database; ICGC, International Cancer Genome Consortium; COSMIC, Catalogue of Somatic Mutations in Cancer; GWAS Catalog, Genome-Wide Association Studies Catalog; GTEx, Genotype-Tissue Expression project; NCBI, National Center for Biotechnology Information; OMIM, Online Mendelian Inheritance in Man; HGNC, HUGO Gene Nomenclature Committee; UniProtKB, Universal Protein Knowledgebase; RVIS, Residual Variation Intolerance Score; LoFtool, Loss-of-Function tool; GDI, Gene Damage Index; pLI score, probability of Loss-of-Function Intolerance; ClinGen, The Clinical Genome Resource; GenCC; Gene Curation Coalition; DECIPHER, Database of Chromosomal Imbalance and Phenotype in Humans using Ensembl Resources; GTR, Genetic Testing Registry; MGI, Mouse Genome Informatics; DGIdb, The Drug Gene Interaction Database; PharmGKB, The Pharmacogenomics Knowledgebase; CTD; The Comparative Toxicogenomics Database.

### Gene-level data source

The basic information, such as gene symbol, gene synonyms, and the location, was sourced from NCBI Gene (68). The functional information was sourced from the Gene Ontology (GO) (69,70), the Universal Protein Knowledgebase (UniProtKB) (71), the InterPro (an integrated database for protein families, domains and functional sites) (72), the NCBI BioSystems database (73) and InBio Map, a scored human protein–protein interaction network (74). Moreover, the quick links of a gene symbol to online databases, including NCBI Gene (68), Online Mendelian Inheritance in Man (OMIM) (75), HUGO Gene Nomenclature Committee (HGNC) (76), Ensembl project (77), and GeneCards (78) were also integrated. Furthermore, we collected the following genic intolerance score of each gene: (I) the residual variation intolerance score (RVIS) (79); (II) the loss-of-function (LoF) intolerance (80); (III) the heptanucleotide context intolerance score (81); (IV) the gene damage index (GDI) (82); (V) the epigenetic cell type deconvolution using single-cell omic references (EPISCORE) (83); (VI) probability of loss-of-function intolerance (pLI) and (VII) the upper bound of 90% confidence interval for observed/expected ratio for LoF variants (LOEUF) (43). Additionally, information related diseases or phenotypes with each gene was curated from various databases: OMIM (75), ClinVar (38), GeneReviews (84), the Clinical Genome Resource (ClinGen) (85), the Human Phenotype Ontology (HPO) (86), the Gene Curation Coalition (GenCC) (87), DECIPHER (a database of genomic variation and phenotype in humans using ensembl resources) (88), the Orphanet database (Orphadata) (89,90), a database of gene-disease associations named DisGeNET (91), the Genetic Testing Registry (GTR) (92), an integrated knowledge database for non-coding RNAs named NONCODE (93), the Mouse Genome Informatics (MGI) (94), and Gene4Denovo (40). Furthermore, we gathered data on gene expression across various tissues from databases such as the Brainspan (95), the Genotype-Tissue Expression (GTEx) project (66), and the Allen Brain Atlases (96) and the protein subcellular location from the Human Protein Atlas (97). Finally, the Drug–Gene Interaction data were sourced from the following databases: the Drug–Gene Interaction database (DGIdb) (98), an online drug information resource named DrugCentral (99), Drug Target Commons (DTC) (100), Pharmacogenomics Knowledgebase (PharmGKB) (101) and Comparative Toxicogenomics Database (CTD) (102) (Table 1).

### Annotation and the conversion of genomic coordinate

Following the approach of VarCards (3), we utilised ANNOVAR (103), an efficient annotation tool, to annotate all SNVs and INDELs (including mitochondrial DNA) using our variant- and gene-level data sources. Additionally, we annotated all curated CNVs using AnnotSV (an integrated tool for CNV annotation) (56). VarCards2 incorporates the genomic coordinates for GRCh37/hg19 and GRCh38/hg38 to facilitate queries. Therefore, for this reason, we employed LiftOver (https://genome.ucsc.edu/cgi-bin/hgLiftOver) to convert one genomic coordinate of some raw data which only provided GRCh37/hg19 or GRCh38/hg38 to the other in this study.

### Database construction and interface

To ensure that users quickly adapt to the functionality of VarCards2, we maintained the simple and popular user interface style characteristic of VarCards. The VarCards2 database was written in Java, JavaScript, Python, and Perl by applying front- and back-end separation models. The back-end was based on Java Spring Boot(https://spring.io/projects/spring-boot), a server-side Java framework that provides services through Application Programming Interface (API) endpoints. The front end, namely the interactive web interface, was powered by the JavaScript libraries Vue (https://vuejs.org) and Element Plus (https://element-plus.org/), which is a Vue 3-based component library for designers and developers that supports all modern browsers across platforms, including Google Chrome, FireFox, Safari, and Microsoft Edge. Annotation of the genomic variants and calculation of all precomputed scores of the genomic variants were performed using Python. The integrated data were stored in a MySQL database, and tab-delimited files were indexed using Tabix (104). The website, database, and search index were deployed on Alibaba Cloud (https://www.alibabacloud.com/).

## Results and web interface

**Figure 1.**
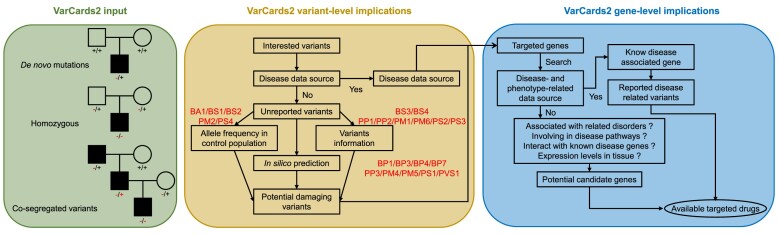
A general workflow of VarCards2. VarCards2 enables the identification of candidate variants from user-uploaded VCF files or through a quick search. For effective prioritization of these variants and the genes associated with genetic diseases, a comprehensive assessment of genomic, genetic, and clinical data sources is imperative. Accordingly, VarCards2 has integrated a range of variant-level and gene-level implications. *Note:* BA1, Benign Stand-alone; BS1/BS2/BS3/BS4, Benign Strong; BP1/BP3/BP4/BP7, Benign Supporting; PP1/PP2/PP3, Pathogenic Supporting; PM1/PM2/PM4/PM5/PM6, Pathogenic Moderate; PS1/PS2/PS3/PS4, Pathogenic Strong; PVS1, Pathogenic Very Strong.

The best practices for offering high-quality services in clinical variant interpretation have been established by the ACMG (2). To streamline genetic counselling in line with the best practices established by the ACMG, VarCards2 integrates a wealth of variant-level and gene-level data sources (Figure 1). In the variant-level section, we include *in silico* predictions, allele frequencies across various populations, information on variants associated with diseases or phenotypes, reported *de novo* mutations and splice variants, and regulatory information such as eQTL, sQTL and epigenomics. In the gene-level section, we offer basic gene information, gene function, associations between genes and diseases or phenotypes, gene expression data, the number of variants in specific genes across diverse populations, and drug-gene interactions. All these features are presented via an intuitive web interface for user convenience.

### Variant-level implications

**Figure 2.**
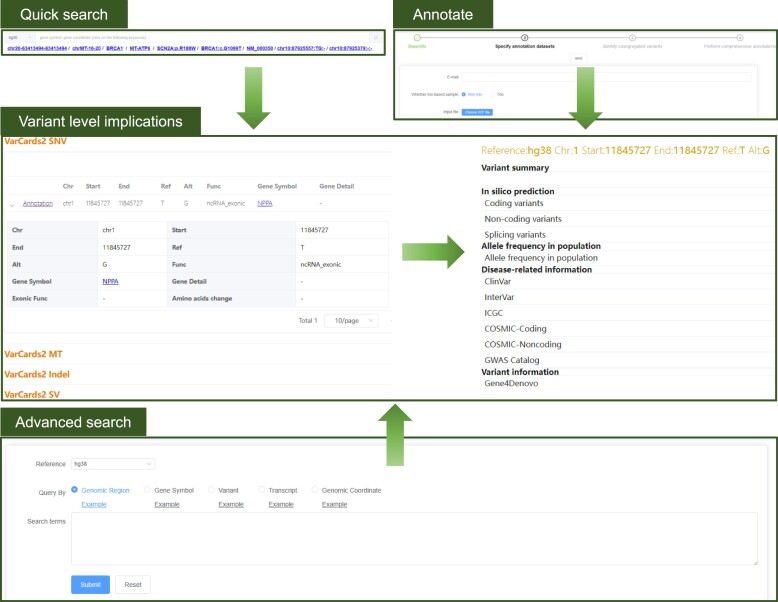
Snapshot of variant-level implications in VarCards2. There are three approaches to access variant-level implications, including ‘Quick search’,‘Advanced search’ and ‘Annotate’. As an example, the results of a quick search for the variant ‘chr1:11845727 T > G (GRCh38)’, including predicted the damaging severity of the variants, allele frequencies in different populations and information in disease related database. VarCards2 offers three methods for accessing variant-level implications: 'Quick search', 'Advanced search' and 'Annotate'. For instance, a quick search for the variant 'chr1:11845727 T > G (GRCh38)' yields results that include the damaging severity of the variant, allele frequencies across various populations, and relevant information from disease-associated databases.

Overall, 8812917339 SNVs, 368820266 INDELs, and 2773555 CNVs in the nuclear genome, and 49704 SNVs and 785 INDELs in the mitochondrial genome were included in VarCards2. When users query rsIDs, genomic positions and regions, gene symbols, genetic variants, or transcript accessions via a quick or advanced search, the search results are displayed in four distinct tables: (I) VarCards2 SNV, (II) VarCards2 MT, (III) VarCards2 INDEL and (IV) VarCards2 CNV. This structured presentation ensures clarity and ease of navigation for users. Each table presents essential details regarding the various types of variants, including chromosomes, reference alleles, alternative alleles, and their impact on amino acids, among other attributes. Upon clicking the "annotation" button in the first column of each table, users are directed to a dedicated page that provides comprehensive functional annotations for the respective variant. The new page displays all variant-level implications, including (I) summary for genetic counselling, (II) *in silico* prediction of function and pathogenicity, (III) AF data sourced from several public population databases, (IV) disease-related information, (V) additional variant insights, such as whether a particular variant is reported as a *de novo* mutation or splicing variant and (VI) regulatory information (Figure 2).

According to the ACMG guidelines, in silico prediction of function and pathogenicity is crucial for determining the potential pathogenicity of a variant. Several criteria, both pathogenic and benign, rely on these predictions, including (I) PVS1, which has a very strong pathogenic weight; (II) PS1, which carries a strong pathogenic weight; (III) PM4 and PM5, with a moderate pathogenic weight; (IV) PP3, with supporting pathogenic weight and (V) BP1, BP3, BP4 and BP7, each with supporting benign weight. To meet the requirements of the above criteria, the number of *in silico* prediction algorithms or tools has been expanded from 23 to 105 compared with its predecessor, VarCards (Supplemental Table 1). These tools cater to various variations, including non-synonymous substitutions, non-coding SNVs, canonical and non-canonical splicing variants, short INDELs, and CNVs. Additionally, AF is a crucial metric according to the ACMG guidelines. If a variant is not detected in several large-scale public population databases, such as gnomAD, 1000genomes, and HRC, this can be considered moderate evidence (PM2) supporting the pathogenicity of the variant. Furthermore, several assessment criteria set by the ACMG guidelines require information regarding other pathogenic variants at identical positions, reported *de novo* mutations, identified splicing sites, and whether the variant is situated on or proximate to a recognised pathogenic or risk gene.

### Gene-level implications

**Figure 3.**
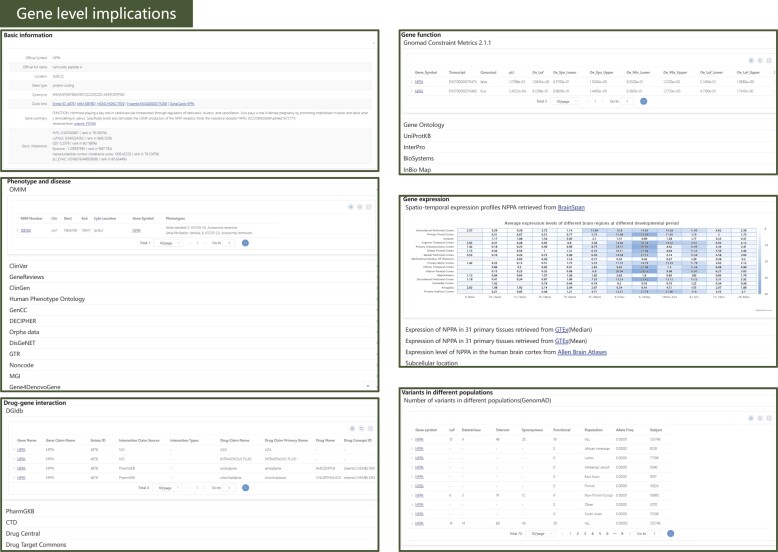
Snapshot of gene-level implications in VarCards2. For instance, details provided for the *NPPA* gene include basic information, gene functions, associated phenotypes and diseases, gene expression patterns, variant distributions across populations, and drug–gene interactions.

In addition to variant-level annotations, VarCards2 offers the corresponding gene-level information to assist with genetic counselling. Gene-level information provided six distinct panels showing annotation details for genes containing or close to the given variant (Figure 3). The 'Basic Information' panel includes details such as: (I) gene names, encompassing the official symbol, full official name, and synonyms sourced from NCBI Gene (68); (II) a summary of the molecular functions of proteins encoded by the specified gene, as sourced from UniProtKB (71); (III) the genetic intolerance score from six studies (43,79–83). The 'Gene Function' panel aggregates information, including GO terms, protein length, mass, subunit structure, domains, biological pathways, gene constraint metrics from gnomAD, and protein-protein interactions corresponding to the protein encoded by the specified gene. The ‘Phenotype and disease’ panel retrieved the reported disease-associated variants or genes from OMIM (75), ClinVar (38), GeneReviews (84), ClinGen (85), HPO (86), GenCC (87), DECIPHER (88), Orphadata (89,90), GTR (92), NONCODE (93), MGI (94) and Gene4Denovo (40). For the ‘Gene expression’ panel, the expression data sourced from Brainspan (95), the GTEx project (66) and the Allen Brain Atlases (96) were illustrated using heatmaps or bar plots separately. Users can view variant counts based on functional effects and observe the overall mutation rates across various populations in the' Variants in Different Populations' panel. For the drug-gene interaction panel, the drugs which affected the given gene were DGIdb (98), DrugCentral (99), DTC (100), PharmGKB (101) and CTD (102). In contrast to their predecessors, VarCards and VarCards2 have enriched their gene-level annotation resources by integrating additional sources such as gene function, gene expression, gene–drug interactions, and phenotype and disease information (Supplemental Table 1).

### Customised annotations

VarCards2 incorporates a feature that allows users to upload genetic data files in the VCF4 format for customised annotations, akin to its predecessor, VarCards. In addition to selecting specific annotations and setting threshold values for in silico prediction scores, VarCards2 not only can pinpoint co-segregated mutations in non-trio-based samples but also can identify *de novo*, homozygous, compound heterozygous, and X-linked hemizygous mutations in trio-based samples. This functionality can be achieved using a straightforward four-step process: (I) users provide an email address to receive annotation results; (II) they choose between the Trio or Non-trio options for the VCF4 data; (III) VCF4 genetic data files are uploaded and (IV) for the Trio option, users must input the sample IDs for the father, mother, and proband, including the proband's gender. If the Non-trio option is selected, users specify the genotype information for each sample, such as heterozygous, homozygous, and wild type.

### Other sections in VarCards2

VarCards2 also provided additional sections, including (I) the upload, which permitted users to upload additional annotation datasets for customised annotations; (II) the data source, which provided a summary of the integrated data sources; (III) the updates, which provided the latest news about VarCards2 and (IV) the tutorial, which provided a further description of VarCards2 and how to use it.

### Case studies

To assess the precision and utility of VarCards2 in detecting a broad range of potential causative variations, we examined several well established and emerging loci based on published literature. (I) For pathogenic SNVs in non-coding regions, we queried chr1:11845727 T > G (GRCh38), located in the 3′ UTR (untranslated regions) of the NPPA gene, which is associated with cardiovascular disorders (105). As we excepted, more than half of the non-coding prediction software categorised this variant as deleterious with a Phred-scaled score ($ - 10{\mathrm{\;}} \times lo{g_{10}}( {rank{\mathrm{\;}}of{\mathrm{\;}}raw{\mathrm{\;}}scores/total{\mathrm{\;}}number{\mathrm{\;}}of{\mathrm{\;}}raw{\mathrm{\;}}scores} )$) >15. According to the ACMG guidelines, this variant has a supporting pathogenic weight in genetic counselling (PP3). Moreover, the variant was not detected in several large-scale public population databases, such as gnomAD, 1000genomes, ExAC and HRC. Therefore, according to the ACMG guidelines, this can be considered a moderate piece of evidence for the pathogenicity (PM2) of the variant. Simultaneously, our gene-level annotation data indicated that NPPA is associated with cardiovascular diseases and is highly expressed in the heart. (II) We examined BBS1:c.G1339A for non-canonical splicing sites. This mutation, a missense variant at a non-canonical splicing site, impairs the splicing process (106). In VarCards2, all 16 splicing-site prediction software tools with available data supported that this site is an alternative splicing site. However, only approximately 20% of the missense mutation prediction tools deem this site detrimental. This underscores the benefits of using diverse prediction software in databases.

## Discussion

It is becoming increasingly evident that variants in the non-coding regions of the human genome significantly impact hereditary diseases (6,7). However, providing clinical interpretations of variants in non-coding areas remains challenging for clinicians and genetic counsellors (4). For general clinicians and genetic counsellors, the optimal approach for interpreting non-coding sequences is to adhere to the ACMG guidelines (8). To facilitate the interpretation of whole-genome sequencing, we incorporated over 150 annotation sources essential for genetic counselling by building VarCards2 within the framework of VarCards. For users seeking additional details, we provide a link that redirects them to the corresponding website for more comprehensive information.

Although many existing tools and databases can annotate non-coding sequences, VarCards2 presents distinct differences (Supplemental Table 2). Compared with seven existing databases, including FAVOR (107), VannoPortal (35), VarSome (108), CADD (10), wAnnovar (109), VEP (110), and SnpEff (111), only VarCards2 could identify co-segregated variants, *de novo* mutations, homozygous variants, compound heterozygous variants, and X-linked hemizygous variants from user-provided VCF files for batch annotation. This feature efficiently assists clinicians and genetic counsellors, who may lack bioinformatics skills, in filtering potential pathogenic variants from extensive data, but also provides evidential support for the interpretation of variant pathogenicity in genetic counselling based on the ACMG guidelines. Furthermore, most existing tools and databases need to encompass comprehensive gene-level annotation. Although VarSome (108) and VEP (110) are exceptions, VarSome (108) operates as a commercial database, whereas VEP (110) only provides linkage information between genes and diseases or phenotypes at the gene level. However, VarCards2 provides users with more than 40 gene-level functional annotations, including 'Gene function’, ‘Gene expression’, 'Gene–drug interaction’, and 'Phenotype and disease information’, through an intuitive web interface for user convenience. Additionally, VarCards2 not only provides the most in silico functional or pathogenic predictions compared to existing databases but is also the only database that offers distinct prediction tools for various types of variants, including SNVs, short INDELs, CNVs, splicing variants and mitochondrial variants. Furthermore, VarCards2 is a unique, non-commercial, one-stop online database capable of providing genetic counselling for SNVs, CNVs, short INDELs and mitochondrial variants. VarCards2 focuses primarily on the clinical interpretation of genetic mutations. It integrates commonly used essential tools and data while discarding less useful and redundant datasets, making it convenient for genetic counselling.

As a comprehensive one-stop online database designed to facilitate genetic counselling, VarCards2 exhibits distinct advantages over the traditional resources used in genetic counselling. For instance, ClinVar (38), an online database, is a valuable and widely used resource for genetic counselling. However, it is important to note that it does not represent all genetic variants owing to its dependency on voluntary submissions. To include as many genetic variants as possible, VarCards2 has not only manually generated close to nine billion SNVs, representing all conceivable SNVs throughout the genome, but has also aggregated reported short INDELs and SVs from a multitude of databases, including dbVAR (42), dbSNP (41), ICGC (37), COSMIC (39), gnomAD (36), Gene4Denovo (40) and ClinVar (38). Additionally, despite ClinVar guidelines, inconsistencies in how different laboratories interpret and classify genetic variants may still arise. This may have led to conflicting classifications of a single variant within the database, and certain submissions may lack comprehensive evidence or interpretations. Consequently, VarCards2 not only aggregates various variant- and gene-level databases for disease information but also provides multiple in silico pathogenic prediction scores and allele frequencies across diverse populations based on the ACMG-AMP guidelines, thereby providing comprehensive evidence to assist users in genetic counselling.

Although VarCards2 offers extensive data to support genetic counselling in line with the ACMG standards and guidelines, users should be aware of the following precautions: First, although we have incorporated over 150 annotation sources into VarCards2, we can only present the datasets used for rating to users, rather than automatically determining them, as this could lead to a high number of false positives. Secondly, because we cannot automate the interpretation and extraction of key information from a large volume of open-access (OA) literature, the vast majority of annotation resources in VarCards2 originate from public databases. Consequently, some crucial information concealed within the most recent publications might be overlooked. Additionally, we encourage users to contribute their in-house annotation datasets because sharing them can benefit a wider user community. Third, disease- and phenotype-related data were collated from several databases, including ClinVar (38), OMIM (75), COSMIC (39) and HPO (86). Consequently, evidence of variations' clinical significance was obtained from diverse teams that employed various criteria and potential methodological biases. Users must remain vigilant of potential false positives in disease- and phenotype-related data (50,112). Furthermore, VarCards2 offers over 100 computational prediction scores for determining the pathogenicity or function of variations, including SNVs, INDELs, and CNVs; users should recognise that these methods vary in their specificity and sensitivity (62,63,113).

Transitioning from VarCards to VarCards2, we refreshed our integrated data sources and incorporated additional datasets vital for the clinical interpretation of non-coding region variants. Although VarCards2 has a vast array of annotation resources, it refrains from directly pinpointing disease-causing variations owing to its intricate genetic testing criteria. However, we are setting sights on enhancing the VarCards2 database during the subsequent phase of automated genetic testing. We also invited the users to share their feedback, suggestions, or valuable data sources. VarCards2 offers a user-friendly gateway for genetic, genomic, and clinical insights into the human genome, expediting the identification and prioritisation of critical variants and genes.

## Supplementary Material

gkad1061_Supplemental_Files

## Data Availability

The data underlying this article are available at http://www.genemed.tech/varcards2/.

## References

[1] Goodwin S. , McPherson J.D., McCombie W.R. Coming of age: ten years of next-generation sequencing technologies. Nat. Rev. Genet. 2016; 17:333–351. 27184599 10.1038/nrg.2016.49 PMC10373632

[2] Richards S. , Aziz N., Bale S., Bick D., Das S., Gastier-Foster J., Grody W.W., Hegde M., Lyon E., Spector E. et al. Standards and guidelines for the interpretation of sequence variants: a joint consensus recommendation of the American College of Medical Genetics and Genomics and the Association for Molecular Pathology. Genet. Med. 2015; 17:405–424. 25741868 10.1038/gim.2015.30 PMC4544753

[3] Li J. , Shi L., Zhang K., Zhang Y., Hu S., Zhao T., Teng H., Li X., Jiang Y., Ji L. et al. VarCards: an integrated genetic and clinical database for coding variants in the human genome. Nucleic Acids Res. 2018; 46:D1039–D1048. 29112736 10.1093/nar/gkx1039 PMC5753295

[4] Zhang F. , Lupski J.R. Non-coding genetic variants in human disease. Hum. Mol. Genet. 2015; 24:R102–R110. 26152199 10.1093/hmg/ddv259 PMC4572001

[5] Elkon R. , Agami R. Characterization of noncoding regulatory DNA in the human genome. Nat. Biotechnol. 2017; 35:732–746. 28787426 10.1038/nbt.3863

[6] Gloss B.S. , Dinger M.E. Realizing the significance of noncoding functionality in clinical genomics. Exp. Mol. Med. 2018; 50:1–8. 10.1038/s12276-018-0087-0 PMC6082831 30089779

[7] French J.D. , Edwards S.L. The role of noncoding variants in heritable disease. Trends Genet. 2020; 36:880–891. 32741549 10.1016/j.tig.2020.07.004

[8] Ellingford J.M. , Ahn J.W., Bagnall R.D., Baralle D., Barton S., Campbell C., Downes K., Ellard S., Duff-Farrier C., FitzPatrick D.R. et al. Recommendations for clinical interpretation of variants found in non-coding regions of the genome. Genome Med. 2022; 14:73. 35850704 10.1186/s13073-022-01073-3 PMC9295495

[9] Giacopuzzi E. , Popitsch N., Taylor J.C. GREEN-DB: a framework for the annotation and prioritization of non-coding regulatory variants from whole-genome sequencing data. Nucleic Acids Res. 2022; 50:2522–2535. 35234913 10.1093/nar/gkac130 PMC8934622

[10] Rentzsch P. , Witten D., Cooper G.M., Shendure J., Kircher M. CADD: predicting the deleteriousness of variants throughout the human genome. Nucleic Acids Res. 2019; 47:D886–D894. 30371827 10.1093/nar/gky1016 PMC6323892

[11] di Iulio J. , Bartha I., Wong E.H.M., Yu H.C., Lavrenko V., Yang D., Jung I., Hicks M.A., Shah N., Kirkness E.F. et al. The human noncoding genome defined by genetic diversity. Nat. Genet. 2018; 50:333–337. 29483654 10.1038/s41588-018-0062-7

[12] Rogers M.F. , Shihab H.A., Gaunt T.R., Campbell C. CScape: a tool for predicting oncogenic single-point mutations in the cancer genome. Sci. Rep. 2017; 7:11597. 28912487 10.1038/s41598-017-11746-4 PMC5599557

[13] Quang D. , Chen Y., Xie X. DANN: a deep learning approach for annotating the pathogenicity of genetic variants. Bioinformatics. 2015; 31:761–763. 25338716 10.1093/bioinformatics/btu703 PMC4341060

[14] Chen L. , Jin P., Qin Z.S. DIVAN: accurate identification of non-coding disease-specific risk variants using multi-omics profiles. Genome Biol. 2016; 17:252. 27923386 10.1186/s13059-016-1112-z PMC5139035

[15] Yang H. , Chen R., Wang Q., Wei Q., Ji Y., Zheng G., Zhong X., Cox N.J., Li B. De novo pattern discovery enables robust assessment of functional consequences of non-coding variants. Bioinformatics. 2019; 35:1453–1460. 30256891 10.1093/bioinformatics/bty826 PMC6499232

[16] Ionita-Laza I. , McCallum K., Xu B., Buxbaum J.D. A spectral approach integrating functional genomic annotations for coding and noncoding variants. Nat. Genet. 2016; 48:214–220. 26727659 10.1038/ng.3477 PMC4731313

[17] Shihab H.A. , Rogers M.F., Gough J., Mort M., Cooper D.N., Day I.N., Gaunt T.R., Campbell C. An integrative approach to predicting the functional effects of non-coding and coding sequence variation. Bioinformatics. 2015; 31:1536–1543. 25583119 10.1093/bioinformatics/btv009 PMC4426838

[18] Rogers M.F. , Shihab H.A., Mort M., Cooper D.N., Gaunt T.R., Campbell C. FATHMM-XF: accurate prediction of pathogenic point mutations via extended features. Bioinformatics. 2018; 34:511–513. 28968714 10.1093/bioinformatics/btx536 PMC5860356

[19] Ioannidis N.M. , Davis J.R., DeGorter M.K., Larson N.B., McDonnell S.K., French A.J., Battle A.J., Hastie T.J., Thibodeau S.N., Montgomery S.B. et al. FIRE: functional inference of genetic variants that regulate gene expression. Bioinformatics. 2017; 33:3895–3901. 28961785 10.1093/bioinformatics/btx534 PMC5860093

[20] Gulko B. , Hubisz M.J., Gronau I., Siepel A. A method for calculating probabilities of fitness consequences for point mutations across the human genome. Nat. Genet. 2015; 47:276–283. 25599402 10.1038/ng.3196 PMC4342276

[21] Gulko B. , Siepel A. An evolutionary framework for measuring epigenomic information and estimating cell-type-specific fitness consequences. Nat. Genet. 2019; 51:335–342. 30559490 10.1038/s41588-018-0300-z PMC6544027

[22] Fu Y. , Liu Z., Lou S., Bedford J., Mu X.J., Yip K.Y., Khurana E., Gerstein M. FunSeq2: a framework for prioritizing noncoding regulatory variants in cancer. Genome Biol. 2014; 15:480. 25273974 10.1186/s13059-014-0480-5 PMC4203974

[23] Lu Q. , Hu Y., Sun J., Cheng Y., Cheung K.H., Zhao H. A statistical framework to predict functional non-coding regions in the human genome through integrated analysis of annotation data. Sci. Rep. 2015; 5:10576. 26015273 10.1038/srep10576 PMC4444969

[24] Huang Y.F. , Gulko B., Siepel A. Fast, scalable prediction of deleterious noncoding variants from functional and population genomic data. Nat. Genet. 2017; 49:618–624. 28288115 10.1038/ng.3810 PMC5395419

[25] Wells A. , Heckerman D., Torkamani A., Yin L., Sebat J., Ren B., Telenti A., di Iulio J. Ranking of non-coding pathogenic variants and putative essential regions of the human genome. Nat. Commun. 2019; 10:5241. 31748530 10.1038/s41467-019-13212-3 PMC6868241

[26] Gussow A.B. , Copeland B.R., Dhindsa R.S., Wang Q., Petrovski S., Majoros W.H., Allen A.S., Goldstein D.B. Orion: detecting regions of the human non-coding genome that are intolerant to variation using population genetics. PLoS One. 2017; 12:e0181604. 28797091 10.1371/journal.pone.0181604 PMC5552289

[27] Zhou L. , Zhao F. Prioritization and functional assessment of noncoding variants associated with complex diseases. Genome Med. 2018; 10:53. 29996888 10.1186/s13073-018-0565-y PMC6042373

[28] Zhang S. , He Y., Liu H., Zhai H., Huang D., Yi X., Dong X., Wang Z., Zhao K., Zhou Y. et al. regBase: whole genome base-wise aggregation and functional prediction for human non-coding regulatory variants. Nucleic Acids Res. 2019; 47:e134. 31511901 10.1093/nar/gkz774 PMC6868349

[29] Smedley D. , Schubach M., Jacobsen J.O.B., Kohler S., Zemojtel T., Spielmann M., Jager M., Hochheiser H., Washington N.L., McMurry J.A. et al. A whole-genome analysis framework for effective identification of pathogenic regulatory variants in Mendelian disease. Am. J. Hum. Genet. 2016; 99:595–606. 27569544 10.1016/j.ajhg.2016.07.005 PMC5011059

[30] Aguet F. , Barbeira A.N., Bonazzola R., Brown A., Castel S.E., Jo B., Kasela S., Kim-Hellmuth S., Liang Y.Y., Parsana P. et al. The GTEx Consortium atlas of genetic regulatory effects across human tissues. Science. 2020; 369:1318–1330. 32913098 10.1126/science.aaz1776 PMC7737656

[31] The ENCODE Project Consortium An integrated encyclopedia of DNA elements in the human genome. Nature. 2012; 489:57–74. 22955616 10.1038/nature11247 PMC3439153

[32] Andersson R. , Gebhard C., Miguel-Escalada I., Hoof I., Bornholdt J., Boyd M., Chen Y., Zhao X., Schmidl C., Suzuki T. et al. An atlas of active enhancers across human cell types and tissues. Nature. 2014; 507:455–461. 24670763 10.1038/nature12787 PMC5215096

[33] Gurbich T.A. , Ilinsky V.V. ClassifyCNV: a tool for clinical annotation of copy-number variants. Sci. Rep. 2020; 10:20375. 33230148 10.1038/s41598-020-76425-3 PMC7683568

[34] Pan Q. , Liu Y.J., Bai X.F., Han X.L., Jiang Y., Ai B., Shi S.S., Wang F., Xu M.C., Wang Y.Z. et al. VARAdb: a comprehensive variation annotation database for human. Nucleic Acids Res. 2021; 49:D1431–D1444. 33095866 10.1093/nar/gkaa922 PMC7779011

[35] Huang D. , Zhou Y., Yi X., Fan X., Wang J., Yao H., Sham P.C., Hao J., Chen K., Li M.J. VannoPortal: multiscale functional annotation of human genetic variants for interrogating molecular mechanism of traits and diseases. Nucleic Acids Res. 2022; 50:D1408–D1416. 34570217 10.1093/nar/gkab853 PMC8728305

[36] Chen S. , Francioli L.C., Goodrich J.K., Collins R.L., Kanai M., Wang Q., Alföldi J., Watts N.A., Vittal C., Gauthier L.D. et al. A genome-wide mutational constraint map quantified from variation in 76,156 human genomes. 2022; bioRxiv doi:21 March 2022, preprint: not peer reviewed 10.1101/2022.03.20.485034.

[37] Zhang J.J. , Bajari R., Andric D., Gerthoffert F., Lepsa A., Nahal-Bose H., Stein L.D., Ferretti V. The International Cancer Genome Consortium Data Portal. Nat. Biotechnol. 2019; 37:367–369. 30877282 10.1038/s41587-019-0055-9

[38] Landrum M.J. , Chitipiralla S., Brown G.R., Chen C., Gu B.S., Hart J., Hoffman D., Jang W., Kaur K., Liu C.L. et al. ClinVar: improvements to accessing data. Nucleic Acids Res. 2020; 48:D835–D844. 31777943 10.1093/nar/gkz972 PMC6943040

[39] Tate J.G. , Bamford S., Jubb H.C., Sondka Z., Beare D.M., Bindal N., Boutselakis H., Cole C.G., Creatore C., Dawson E. et al. COSMIC: the Catalogue Of Somatic Mutations In Cancer. Nucleic Acids Res. 2019; 47:D941–D947. 30371878 10.1093/nar/gky1015 PMC6323903

[40] Zhao G. , Li K., Li B., Wang Z., Fang Z., Wang X., Zhang Y., Luo T., Zhou Q., Wang L. et al. Gene4Denovo: an integrated database and analytic platform for de novo mutations in humans. Nucleic Acids Res. 2020; 48:D913–D926. 31642496 10.1093/nar/gkz923 PMC7145562

[41] Sherry S.T. , Ward M.H., Kholodov M., Baker J., Phan L., Smigielski E.M., Sirotkin K. dbSNP: the NCBI database of genetic variation. Nucleic Acids Res. 2001; 29:308–311. 11125122 10.1093/nar/29.1.308 PMC29783

[42] Lappalainen I. , Lopez J., Skipper L., Hefferon T., Spalding J.D., Garner J., Chen C., Maguire M., Corbett M., Zhou G. et al. dbVar and DGVa: public archives for genomic structural variation. Nucleic Acids Res. 2013; 41:D936–D941. 23193291 10.1093/nar/gks1213 PMC3531204

[43] Karczewski K.J. , Francioli L.C., Tiao G., Cummings B.B., Alfoldi J., Wang Q., Collins R.L., Laricchia K.M., Ganna A., Birnbaum D.P. et al. The mutational constraint spectrum quantified from variation in 141,456 humans. Nature. 2020; 581:434–443. 32461654 10.1038/s41586-020-2308-7 PMC7334197

[44] Lek M. , Karczewski K.J., Minikel E.V., Samocha K.E., Banks E., Fennell T., O’Donnell-Luria A.H., Ware J.S., Hill A.J., Cummings B.B. et al. Analysis of protein-coding genetic variation in 60,706 humans. Nature. 2016; 536:285–291. 27535533 10.1038/nature19057 PMC5018207

[45] Altshuler D.M. , Durbin R.M., Abecasis G.R., Bentley D.R., Chakravarti A., Clark A.G., Donnelly P., Eichler E.E., Flicek P., Gabriel S.B. et al. A global reference for human genetic variation. Nature. 2015; 526:68–74. 26432245 10.1038/nature15393 PMC4750478

[46] Fu W. , O’Connor T.D., Jun G., Kang H.M., Abecasis G., Leal S.M., Gabriel S., Rieder M.J., Altshuler D., Shendure J. et al. Analysis of 6,515 exomes reveals the recent origin of most human protein-coding variants. Nature. 2013; 493:216–220. 23201682 10.1038/nature11690 PMC3676746

[47] Glusman G. , Caballero J., Mauldin D.E., Hood L., Roach J.C. Kaviar: an accessible system for testing SNV novelty. Bioinformatics. 2011; 27:3216–3217. 21965822 10.1093/bioinformatics/btr540 PMC3208392

[48] McCarthy S. , Das S., Kretzschmar W., Delaneau O., Wood A.R., Teumer A., Kang H.M., Fuchsberger C., Danecek P., Sharp K. et al. A reference panel of 64,976 haplotypes for genotype imputation. Nat. Genet. 2016; 48:1279–1283. 27548312 10.1038/ng.3643 PMC5388176

[49] Lott M.T. , Leipzig J.N., Derbeneva O., Xie H.M., Chalkia D., Sarmady M., Procaccio V., Wallace D.C. mtDNA Variation and Analysis Using Mitomap and Mitomaster. Curr. Protoc. Bioinformatics. 2013; 44:1.23.1–1.23.26. 10.1002/0471250953.bi0123s44 PMC4257604 25489354

[50] Li Q. , Wang K. InterVar: clinical interpretation of genetic variants by the 2015 ACMG-AMP guidelines. Am. J. Hum. Genet. 2017; 100:267–280. 28132688 10.1016/j.ajhg.2017.01.004 PMC5294755

[51] Sollis E. , Mosaku A., Abid A., Buniello A., Cerezo M., Gil L., Groza T., Gunes O., Hall P., Hayhurst J. et al. The NHGRI-EBI GWAS Catalog: knowledgebase and deposition resource. Nucleic Acids Res. 2022; 51:D977–D985. 10.1093/nar/gkac1010 PMC9825413 36350656

[52] Kircher M. , Witten D.M., Jain P., O’Roak B.J., Cooper G.M., Shendure J. A general framework for estimating the relative pathogenicity of human genetic variants. Nat. Genet. 2014; 46:310–315. 24487276 10.1038/ng.2892 PMC3992975

[53] Li S. , van der Velde K.J., de Ridder D., van Dijk A.D.J., Soudis D., Zwerwer L.R., Deelen P., Hendriksen D., Charbon B., van Gijn M.E. et al. CAPICE: a computational method for Consequence-Agnostic Pathogenicity Interpretation of Clinical Exome variations. Genome Medicine. 2020; 12:75. 32831124 10.1186/s13073-020-00775-w PMC7446154

[54] Ferlaino M. , Rogers M.F., Shihab H.A., Mort M., Cooper D.N., Gaunt T.R., Campbell C. An integrative approach to predicting the functional effects of small indels in non-coding regions of the human genome. BMC Bioinf. 2017; 18:442. 10.1186/s12859-017-1862-y PMC5955213 28985712

[55] Choi Y. , Sims G.E., Murphy S., Miller J.R., Chan A.P. Predicting the functional effect of amino acid substitutions and indels. PLoS One. 2012; 7:e46688. 23056405 10.1371/journal.pone.0046688 PMC3466303

[56] Geoffroy V. , Herenger Y., Kress A., Stoetzel C., Piton A., Dollfus H., Muller J. AnnotSV: an integrated tool for structural variations annotation. Bioinformatics. 2018; 34:3572–3574. 29669011 10.1093/bioinformatics/bty304

[57] Kleinert P. , Kircher M. A framework to score the effects of structural variants in health and disease. Genome Res. 2022; 32:766–777. 35197310 10.1101/gr.275995.121 PMC8997355

[58] Sharo A.G. , Hu Z.Q., Sunyaev S.R., Brenner S.E. StrVCTVRE: a supervised learning method to predict the pathogenicity of human genome structural variants. Am. J. Hum. Genet. 2022; 109:195–209. 35032432 10.1016/j.ajhg.2021.12.007 PMC8874149

[59] Liu X.M. , Li C., Mou C.C., Dong Y.B., Tu Y.C. dbNSFP v4: a comprehensive database of transcript-specific functional predictions and annotations for human nonsynonymous and splice-site SNVs. Genome Med. 2020; 12:103. 33261662 10.1186/s13073-020-00803-9 PMC7709417

[60] Liu X. , Jian X., Boerwinkle E. dbNSFP: a lightweight database of human nonsynonymous SNPs and their functional predictions. Hum. Mutat. 2011; 32:894–899. 21520341 10.1002/humu.21517 PMC3145015

[61] Cheng J. , Novati G., Pan J., Bycroft C., Žemgulytė A., Applebaum T., Pritzel A., Wong L.H., Zielinski M., Sargeant T. et al. Accurate proteome-wide missense variant effect prediction with AlphaMissense. Science. 2023; 381:1284–1285. 37733863 10.1126/science.adg7492

[62] Wang Z. , ZhaoG., LiB., FangZ., ChenQ., WangX., LuoT., WangY., ZhouQ., LiK.et al. Performance comparison of computational methods for the prediction of the function and pathogenicity of non-coding variants. Genomics Proteomics Bioinformatics.2022; 10.1016/j.gpb.2022.02.002.35272052

[63] Li K.K. , Luo T.F., Zhu Y., Huang Y.F., Wang A., Zhang D., Dong L.J., Wang Y.J., Wang R., Tang D.D. et al. Performance evaluation of differential splicing analysis methods and splicing analytics platform construction. Nucleic Acids Res. 2022; 50:9115–9126. 35993808 10.1093/nar/gkac686 PMC9458456

[64] Castellana S. , Biagini T., Petrizzelli F., Parca L., Panzironi N., Caputo V., Vescovi A.L., Carella M., Mazza T. MitImpact 3: modeling the residue interaction network of the respiratory chain subunits. Nucleic Acids Res. 2021; 49:D1282–D1288. 33300029 10.1093/nar/gkaa1032 PMC7779045

[65] Castellana S. , Ronai J., Mazza T. MitImpact: an exhaustive collection of pre-computed pathogenicity predictions of human mitochondrial non-synonymous variants. Hum. Mutat. 2015; 36:E2413–E2422. 25516408 10.1002/humu.22720

[66] GTEx Consortium The Genotype-Tissue Expression (GTEx) project. Nat. Genet. 2013; 45:580–585. 23715323 10.1038/ng.2653 PMC4010069

[67] Boix C.A. , James B.T., Park Y.P., Meuleman W., Kellis M. Regulatory genomic circuitry of human disease loci by integrative epigenomics. Nature. 2021; 590:300–307. 33536621 10.1038/s41586-020-03145-z PMC7875769

[68] Brown G.R. , Hem V., Katz K.S., Ovetsky M., Wallin C., Ermolaeva O., Tolstoy I., Tatusova T., Pruitt K.D., Maglott D.R. et al. Gene: a gene-centered information resource at NCBI. Nucleic Acids Res. 2015; 43:D36–D42. 25355515 10.1093/nar/gku1055 PMC4383897

[69] Ashburner M. , Ball C.A., Blake J.A., Botstein D., Butler H., Cherry J.M., Davis A.P., Dolinski K., Dwight S.S., Eppig J.T. et al. Gene Ontology: tool for the unification of biology. Nat. Genet. 2000; 25:25–29. 10802651 10.1038/75556 PMC3037419

[70] Aleksander S.A. , Balhoff J., Carbon S., Cherry J.M., Drabkin H.J., Ebert D., Feuermann M., Gaudet P., Harris N.L., Hill D.P. et al. The Gene Ontology knowledgebase in 2023. Genetics. 2023; 224:iyad031. 36866529 10.1093/genetics/iyad031 PMC10158837

[71] Bateman A. , Martin M.J., Orchard S., Magrane M., Ahmad S., Alpi E., Bowler-Barnett E.H., Britto R., Cukura A., Denny P. et al. UniProt: the Universal Protein Knowledgebase in 2023. Nucleic Acids Res. 2022; 51:D523–D531. 10.1093/nar/gkac1052 PMC9825514 36408920

[72] Paysan-Lafosse T. , Blum M., Chuguransky S., Grego T., Pinto B.L., Salazar G.A., Bileschi M.L., Bork P., Bridge A., Colwell L. et al. InterPro in 2022. Nucleic Acids Res. 2022; 51:D418–D427. 10.1093/nar/gkac993 PMC9825450 36350672

[73] Geer L.Y. , Marchler-Bauer A., Geer R.C., Han L., He J., He S., Liu C., Shi W., Bryant S.H. The NCBI BioSystems database. Nucleic Acids Res. 2009; 38:D492–D496. 19854944 10.1093/nar/gkp858 PMC2808896

[74] Li T.B. , Wernersson R., Hansen R.B., Horn H., Mercer J., Slodkowicz G., Workman C.T., Rigina O., Rapacki K., Staerfeldt H.H. et al. A scored human protein-protein interaction network to catalyze genomic interpretation. Nat. Methods. 2017; 14:61–64. 27892958 10.1038/nmeth.4083 PMC5839635

[75] Amberger J.S. , Bocchini C.A., Scott A.F., Hamosh A. OMIM.org: leveraging knowledge across phenotype-gene relationships. Nucleic Acids Res. 2019; 47:D1038–D1043. 30445645 10.1093/nar/gky1151 PMC6323937

[76] Seal R.L. , Braschi B., Gray K., Jones T.E.M., Tweedie S., Haim-Vilmovsky L., Bruford E.A. Genenames.org: the HGNC resources in 2023. Nucleic Acids Res. 2022; 51:D1003–D1009. 10.1093/nar/gkac888 PMC9825485 36243972

[77] Cunningham F. , Allen J.E., Allen J., Alvarez-Jarreta J., Amode M.R., Armean I.M., Austine-Orimoloye O., Azov A.G., Barnes I., Bennett R. et al. Ensembl 2022. Nucleic Acids Res. 2021; 50:D988–D995. 10.1093/nar/gkab1049 PMC8728283 34791404

[78] Stelzer G. , Rosen N., Plaschkes I., Zimmerman S., Twik M., Fishilevich S., Stein T.I., Nudel R., Lieder I., Mazor Y. et al. The GeneCards Suite: from Gene Data Mining to Disease Genome Sequence Analyses. Curr. Protoc. Bioinformatics. 2016; 54:1.30.1–1.30.33. 10.1002/cpbi.5 27322403

[79] Petrovski S. , Gussow A.B., Wang Q.L., Halvorsen M., Han Y.J., Weir W.H., Allen A.S., Goldstein D.B. The intolerance of regulatory sequence to genetic variation predicts gene dosage sensitivity. PLoS Genet. 2015; 11:e1005492. 26332131 10.1371/journal.pgen.1005492 PMC4557908

[80] Fadista J. , Oskolkov N., Hansson O., Groop L. LoFtool: a gene intolerance score based on loss-of-function variants in 60 706 individuals. Bioinformatics. 2017; 33:471–474. 27563026 10.1093/bioinformatics/btv602

[81] Aggarwala V. , Voight B.F. An expanded sequence context model broadly explains variability in polymorphism levels across the human genome. Nat. Genet. 2016; 48:349–355. 26878723 10.1038/ng.3511 PMC4811712

[82] Itan Y. , Shang L., Boisson B., Patin E., Bolze A., Moncada-Velez M., Scott E., Ciancanelli M.J., Lafaille F.G., Markle J.G. et al. The human gene damage index as a gene-level approach to prioritizing exome variants. Proc. Natl. Acad. Sci. U.S.A. 2015; 112:13615–13620. 26483451 10.1073/pnas.1518646112 PMC4640721

[83] Teschendorff A.E. , Zhu T.Y., Breeze C.E., Beck S. EPISCORE: cell type deconvolution of bulk tissue DNA methylomes from single-cell RNA-Seq data. Genome Biol. 2020; 21:221. 32883324 10.1186/s13059-020-02126-9 PMC7650528

[84] Adam M.P. , Mirzaa G.M., Pagon R.A., Wallace S.E., Bean L.J.H., Gripp K.W., Amemiya A. GeneReviews^®^. 1993; Seattle, WA University of Washington. 32027476

[85] Rehm H.L. , Berg J.S., Brooks L.D., Bustamante C.D., Evans J.P., Landrum M.J., Ledbetter D.H., Maglott D.R., Martin C.L., Nussbaum R.L. et al. ClinGen — The Clinical Genome Resource. N. Engl. J. Med. 2015; 372:2235–2242. 26014595 10.1056/NEJMsr1406261 PMC4474187

[86] Kohler S. , Gargano M., Matentzoglu N., Carmody L.C., Lewis-Smith D., Vasilevsky N.A., Danis D., Balagura G., Baynam G., Brower A.M. et al. The Human Phenotype Ontology in 2021. Nucleic Acids Res. 2021; 49:D1207–D1217. 33264411 10.1093/nar/gkaa1043 PMC7778952

[87] DiStefano M.T. , Goehringer S., Babb L., Alkuraya F.S., Amberger J., Amin M., Austin-Tse C., Balzotti M., Berg J.S., Birney E. et al. The Gene Curation Coalition: a global effort to harmonize gene–disease evidence resources. Genet. Med. 2022; 24:1732–1742. 35507016 10.1016/j.gim.2022.04.017 PMC7613247

[88] Firth H.V. , Richards S.M., Bevan A.P., Clayton S., Corpas M., Rajan D., Van Vooren S., Moreau Y., Pettett R.M., Carter N.P. DECIPHER: database of Chromosomal Imbalance and Phenotype in Humans Using Ensembl Resources. Am. J. Hum. Genet. 2009; 84:524–533. 19344873 10.1016/j.ajhg.2009.03.010 PMC2667985

[89] Pavan S. , Rommel K., Marquina M.E.M., Hohn S., Lanneau V., Rath A. Clinical practice guidelines for rare diseases: the Orphanet Database. PLoS One. 2017; 12:e0170365. 28099516 10.1371/journal.pone.0170365 PMC5242437

[90] Wakap S.N. , Lambert D.M., Olry A., Rodwell C., Gueydan C., Lanneau V., Mury D., Le Cam Y., Rath A. Estimating cumulative point prevalence of rare diseases: analysis of the Orphanet database. Eur. J. Hum. Genet. 2020; 28:165–173. 31527858 10.1038/s41431-019-0508-0 PMC6974615

[91] Pinero J. , Sauch J., Sanz F., Furlong L.I. The DisGeNET cytoscape app: exploring and visualizing disease genomics data. Comput. Struct. Biotechnol. J. 2021; 19:2960–2967. 34136095 10.1016/j.csbj.2021.05.015 PMC8163863

[92] Rubinstein W.S. , Maglott D.R., Lee J.M., Kattman B.L., Malheiro A.J., Ovetsky M., Hem V., Gorelenkov V., Song G.F., Wallin C. et al. The NIH genetic testing registry: a new, centralized database of genetic tests to enable access to comprehensive information and improve transparency. Nucleic Acids Res. 2013; 41:D925–D935. 23193275 10.1093/nar/gks1173 PMC3531155

[93] Zhao L.H. , Wang J.J., Li Y.Y., Song T.R., Wu Y., Fang S.S., Bu D.C., Li H., Sun L., Pei D. et al. NONCODEV6: an updated database dedicated to long non-coding RNA annotation in both animals and plants. Nucleic Acids Res. 2021; 49:D165–D171. 33196801 10.1093/nar/gkaa1046 PMC7779048

[94] Blake J.A. , Baldarelli R., Kadin J.A., Richardson J.E., Smith C.L., Bult C.J., Grp M.G.D. Mouse Genome Database (MGD): knowledgebase for mouse-human comparative biology. Nucleic Acids Res. 2021; 49:D981–D987. 33231642 10.1093/nar/gkaa1083 PMC7779030

[95] Miller J.A. , Ding S.L., Sunkin S.M., Smith K.A., Ng L., Szafer A., Ebbert A., Riley Z.L., Royall J.J., Aiona K. et al. Transcriptional landscape of the prenatal human brain. Nature. 2014; 508:199–206. 24695229 10.1038/nature13185 PMC4105188

[96] Sunkin S.M. , Ng L., Lau C., Dolbeare T., Gilbert T.L., Thompson C.L., Hawrylycz M., Dang C. Allen Brain Atlas: an integrated spatio-temporal portal for exploring the central nervous system. Nucleic Acids Res. 2013; 41:D996–D1008. 23193282 10.1093/nar/gks1042 PMC3531093

[97] Thul P.J. , Åkesson L., Wiking M., Mahdessian D., Geladaki A., Ait Blal H., Alm T., Asplund A., Björk L., Breckels L.M. et al. A subcellular map of the human proteome. Science. 2017; 356:eaal3321. 28495876 10.1126/science.aal3321

[98] Freshour S.L. , Kiwala S., Cotto K.C., Coffman A.C., McMichael J.F., Song J.J., Griffith M., Griffith O.L., Wagner A.H. Integration of the Drug–Gene Interaction Database (DGIdb 4.0) with open crowdsource efforts. Nucleic Acids Res. 2021; 49:D1144–D1151. 33237278 10.1093/nar/gkaa1084 PMC7778926

[99] Avram S. , Wilson T.B., Curpan R., Halip L., Borota A., Bora A., Bologa C.G., Holmes J., Knockel J., Yang J.J. et al. DrugCentral 2023 extends human clinical data and integrates veterinary drugs. Nucleic Acids Res. 2022; 51:D1276–D1287. 10.1093/nar/gkac1085 PMC9825566 36484092

[100] Tang J. , Tanoli Z.U.R., Ravikumar B., Alam Z., Rebane A., Vaha-Koskela M., Peddinti G., van Adrichem A.J., Wakkinen J., Jaiswal A. et al. Drug Target Commons: a Community Effort to Build a Consensus Knowledge Base for Drug-Target Interactions. Cell Chem Biol. 2018; 25:224–229. 29276046 10.1016/j.chembiol.2017.11.009 PMC5814751

[101] Whirl-Carrillo M. , Huddart R., Gong L., Sangkuhl K., Thorn C.F., Whaley R., Klein T.E. 2021) An evidence-based framework for evaluating pharmacogenomics knowledge for personalized medicine. Clin. Pharmacol. Ther. 110:563–572. 34216021 10.1002/cpt.2350 PMC8457105

[102] Davis A.P. , Wiegers T.C., Johnson R.J., Sciaky D., Wiegers J., Mattingly C.J. Comparative Toxicogenomics Database (CTD): update 2023. Nucleic Acids Res. 2022; 51:D1257–D1262. 10.1093/nar/gkac833 PMC9825590 36169237

[103] Wang K. , Li M., Hakonarson H. ANNOVAR: functional annotation of genetic variants from high-throughput sequencing data. Nucleic Acids Res. 2010; 38:e164. 20601685 10.1093/nar/gkq603 PMC2938201

[104] Li H. Tabix: fast retrieval of sequence features from generic TAB-delimited files. Bioinformatics. 2011; 27:718–719. 21208982 10.1093/bioinformatics/btq671 PMC3042176

[105] Johnson R. , Richter N., Bogu G.K., Bhinge A., Teng S.W., Choo S.H., Andrieux L.O., de Benedictis C., Jauch R., Stanton L.W. A genome-wide screen for genetic variants that modify the recruitment of REST to its target genes. PLos Genet. 2012; 8:128–140. 10.1371/journal.pgen.1002624 PMC3320604 22496669

[106] Yan K. , Sun Y., Yang Y., Liu B., Dong M. Case report: identification pathogenic abnormal splicing of BBS1 causing Bardet-Biedl Syndrome Type I (BBS1) due to missense mutation. Front. Genet. 2022; 13:849562. 35692835 10.3389/fgene.2022.849562 PMC9186647

[107] Zhou H. , Arapoglou T., Li X., Li Z., Zheng X., Moore J., Asok A., Kumar S., Blue E.E., Buyske S. et al. FAVOR: functional annotation of variants online resource and annotator for variation across the human genome. Nucleic Acids Res. 2023; 51:D1300–D1311. 36350676 10.1093/nar/gkac966 PMC9825437

[108] Kopanos C. , Tsiolkas V., Kouris A., Chapple C.E., Aguilera M.A., Meyer R., Massouras A. VarSome: the human genomic variant search engine. Bioinformatics. 2019; 35:1978–1980. 30376034 10.1093/bioinformatics/bty897 PMC6546127

[109] Chang X. , Wang K. wANNOVAR: annotating genetic variants for personal genomes via the web. J. Med. Genet. 2012; 49:433–436. 22717648 10.1136/jmedgenet-2012-100918 PMC3556337

[110] McLaren W. , Gil L., Hunt S.E., Riat H.S., Ritchie G.R.S., Thormann A., Flicek P., Cunningham F. The Ensembl variant effect predictor. Genome Biol. 2016; 17:122. 27268795 10.1186/s13059-016-0974-4 PMC4893825

[111] Cingolani P. , Platts A., Wang L.L., Coon M., Nguyen T., Wang L., Land S.J., Lu X.Y., Ruden D.M. A program for annotating and predicting the effects of single nucleotide polymorphisms, SnpEff. fly. 2012; 6:80–92. 22728672 10.4161/fly.19695 PMC3679285

[112] Shearer A.E. , Eppsteiner R.W., Booth K.T., Ephraim S.S., Gurrola J., Simpson A., Black-Ziegelbein E.A., Joshi S., Ravi H., Giuffre A.C. et al. Utilizing ethnic-specific differences in minor allele frequency to recategorize reported pathogenic deafness variants. Am. J. Hum. Genet. 2014; 95:445–453. 25262649 10.1016/j.ajhg.2014.09.001 PMC4185121

[113] Li J. , Zhao T., Zhang Y., Zhang K., Shi L., Chen Y., Wang X., Sun Z. Performance evaluation of pathogenicity-computation methods for missense variants. Nucleic Acids Res. 2018; 46:7793–7804. 30060008 10.1093/nar/gky678 PMC6125674

