# Supplementary material for: VarCards2: an integrated genetic and clinical database for ACMG-AMP variant-interpretation guidelines in the human whole genome
Source: Nucleic Acids Res. 2023 Nov 13;52(D1):D1478–89. doi: 10.1093/nar/gkad1061 (PMC10767961; doi:10.1093/nar/gkad1061)
Supplement: gkad1061_Supplemental_Files [file gkad1061_supplemental_files.zip › Supplemental Table 1.docx]

**Supplemental Table1. Statistical Insights into the Enhancements from VarCards to VarCards2**

|  | VarCards | VarCards2 |
| --- | --- | --- |
| **The number of variants in nuclear genome** | | |
| Artificially generated SNVs | 110,154,363 | 8,812,917,339 |
| Reported small INDELs | 1,223,370 | 368,820,266 |
| Reported CNVs | 0 | 2,773,555 |
| **The number of variants in mitochondrial genome** | | |
| Artificially generated SNVs | 0 | 49,704 |
| Reported small INDELs | 0 | 785 |
| **Variation-level implication** | | |
| Predictive algorithms for coding region | 23 | 50 |
| Predictive algorithms for non-coding region | 0 | 24 |
| Predictive algorithms for splice variants | 0 | 19 |
| Predictive algorithms for small indels | 2 | 4 |
| Predictive algorithms for cnvs | 0 | 4 |
| Predictive algorithms for mitochondrial genome | 0 | 25 |
| **Gene-level implication** | | |
| Gene function | 5 | 6 |
| Phenotype and disease | 5 | 13 |
| Gene expression | 2 | 4 |
| Target drug databases | 1 | 5 |
