# Supplementary material for: VarCards2: an integrated genetic and clinical database for ACMG-AMP variant-interpretation guidelines in the human whole genome
Source: Nucleic Acids Res. 2023 Nov 13;52(D1):D1478–89. doi: 10.1093/nar/gkad1061 (PMC10767961; doi:10.1093/nar/gkad1061)
Supplement: gkad1061_Supplemental_Files [file gkad1061_supplemental_files.zip › Supplemental Table 2.docx]

**Supplemental Table 2. Comparison of VarCards2 and other functional annotation tools.**

|  | VarCards2 | FAVOR | VannoPortal | VarSome | CADD | wAnnovar | VEP | SnpEff |
| --- | --- | --- | --- | --- | --- | --- | --- | --- |
| **Website** | √ | √ | √ | √ | √ | √ | √ | × |
| **Organize annotations in functional blocks for navigation** | √ | √ | √ | √ | × | × | × | × |
| **Results display on website** | √ | √ | √ | √ | √ | √ | √ | × |
| **Annotation of all possible 9 billion SNVs** | √ | √ | × | × | √ | √ | √ | √ |
| **Large input support** | √ | √ | × | × | × | √ | √ | √ |
| **Variant annotation function** | √ | √ | × | × | × | √ | √ | × |
| **Gene-level (Gene function)** | √ | × | × | √ | × | × | × | × |
| **Gene-level (Phenotype and disease)** | √ | × | × | √ | × | × | √ | × |
| **Gene-level (Gene expression)** | √ | × | × | √ | × | × | × | × |
| **Gene-level (Variants in different population)** | √ | × | × | √ | × | × | × | × |
| **Gene-level (Drug-gene interaction)** | √ | × | × | √ | × | × | × | × |
| **Variant-level (SNP)** | √ | √ | √ | √ | √ | √ | √ | √ |
| **Variant-level (MT)** | √ | × | × | √ | × | √ | √ | √ |
| **Variant-level (Indel)** | √ | √ | √ | √ | √ | √ | √ | √ |
| **Variant-level (CNV)** | √ | × | × | √ | × | × | × | × |
| **hg19 and hg38 build** | √ | × | √ | √ | √ | √ | √ | √ |
| **search feature (Variant Position** | √ | √ | √ | √ | √ | √ | √ | × |
| **search feature (rsID)** | √ | √ | √ | √ | × | × | √ | × |
| **search feature (Region)** | √ | √ | × | √ | √ | √ | √ | × |
| **search feature (Gene Symbol)** | √ | √ | × | √ | × | × | × | × |
| **search feature (HGVS)** | √ | × | √ | √ | × | × | √ | × |
| **search feature (Transcript)** | √ | × | × | √ | × | × | × | × |
| **identify the co-segregated variants (non-trio-based samples)** | √ | × | × | × | × | × | × | × |
| **identify the *De-novo* variants (trio-based samples)** | √ | × | × | × | × | × | × | × |
| **identify the homozygous variants (trio-based samples)** | √ | × | × | × | × | × | × | × |
| **identify the compound heterozygous variants (trio-based samples)** | √ | × | × | × | × | × | × | × |
| **identify the X-linked hemizygous variants (trio-based samples)** | √ | × | × | × | × | × | × | × |
| **Number of coding SNVs pathogenicity prediction algorithms** | 50 | 47 | 47 | 47 | 12 | 47 | 47 | × |
| **Number of non-coding SNVs pathogenicity prediction algorithms** | 24 | 9 | 21 | 9 | × | 9 | 9 | × |
| **Number of indels pathogenicity prediction algorithms** | 4 | 2 | 2 | 2 | × | 2 | 2 | × |
| **Number of splicing variants pathogenicity prediction algorithms** | 19 | × | 6 | 2 | 4 | × | 4 | × |
| **Number of mitochondrial variants pathogenicity prediction algorithms** | 25 | × | × | 12 | × | 9 | 9 | × |
| **Number of copy number variations pathogenicity prediction algorithms** | 4 | × | × | × | × | × | × | × |
